# Genomic and Metabolomic Insights into Metabolites of a *Streptomyces* Isolate Associated with *Chromodoris quadricolor*, a Red Sea Nudibranch

**DOI:** 10.3390/md23100404

**Published:** 2025-10-17

**Authors:** Samar M. Abdelrahman, Zoe A. Pratte, Manar El Samak, Noura S. Dosoky, Amro M. S. Hanora, Frank J. Stewart, Nicole B. Lopanik

**Affiliations:** 1 School of Earth and Atmospheric Sciences, Georgia Institute of Technology, Atlanta, GA 30332, USA; nicole.lopanik@cancer.org; 2 Department of Botany and Microbiology, Faculty of Science, Suez University, Suez 43221, Egypt; 3 School of Biological Sciences, Georgia Institute of Technology, Atlanta, GA 30332, USA; zoe.pratte@montana.edu (Z.A.P.); frank.stewart@montana.edu (F.J.S.); 4 Department of Microbiology & Immunology, Montana State University, Bozeman, MT 59717, USA; 5 Department of Microbiology & Immunology, Faculty of Pharmacy, Suez Canal University, Ismailia 43518, Egypt; manarelsamak@yahoo.com (M.E.S.); ahanora@yahoo.com (A.M.S.H.); 6 Salt Lake Community College, Salt Lake City, UT 84123, USA; nahmed8@slcc.edu; 7 American Cancer Society, Atlanta, GA 30303, USA

**Keywords:** actinomycetes, *Streptomyces*, genomics, biosynthetic gene cluster, metabolomics, HPLC-HRMS/MS

## Abstract

The marine invertebrate-associated microbiome has garnered significant interest in recent years due to its wealth of novel genes that can be explored for biomining. By combining genomics with untargeted data-dependent mass spectrometry (MS) and molecular networking, we characterized the secreted metabolome of *Streptomyces* sp. In a previous study, we isolated and characterized a strain of *Streptomyces*, designated as strain 34, from the nudibranch *Chromodoris quadricolor*, collected by SCUBA diving in the Red Sea near El Tor in the Gulf of Suez, Egypt. In the present study, the *Streptomyces* isolate was identified as *Streptomyces tunisiensis* GCF 039538125 1 (*p*-value: 0). Genomic and metabolomic analysis reveal 36 predicted biosynthetic gene clusters. A total of 569 metabolites were detected in the culture, with 86 of these being identified based on standards and public spectral libraries. Moreover, a single lassopeptide synthesis gene cluster was found in both the genome and the metabolic extract, along with various sets of siderophores identified in the metabolic extract. Since the metabolic processes of marine invertebrate microbiomes are poorly understood, our findings are a significant addition to the research on metabolism in host microbiomes.

## 1. Introduction

Natural products of microbial origin have garnered significant attention due to their diverse chemical structures and bioactivity, accounting for nearly 35% of all currently available drugs [1]. Moreover, natural products play an important role in drug discovery for cancer and infectious diseases [2]. However, the use of antibiotics presents considerable challenges for medical practitioners and researchers because of the rise and spread of antibiotic-resistant pathogens. Additionally, the low success rate of antibiotic drug discovery programs worldwide has led to a critical shortage of new antibiotic drug candidates [3,4].

Marine ecosystems are considered a valuable source of bioactive compounds due to the rich biodiversity within their animal and microbial communities [5,6]. Nudibranchs, marine invertebrates that lack a shell, are covered by a layer of mucus containing high levels of proteins and lipids, and are exposed to pathogenic microorganisms [7]. Research has revealed that many nudibranchs produce defensive secondary metabolites to protect themselves from such pathogens [8,9]. Our findings from a previous study suggest that the diverse microorganisms associated with nudibranchs play a significant role in the production of these secondary metabolites [10].

*Streptomyces* species are recognized as a major source of a wide variety of natural compounds due to their complex secondary metabolism [11,12,13]. These microorganisms produce approximately 100,000 antibiotic substances, which account for 70–80% of all naturally occurring bioactive compounds used in pharmacology and agriculture. This genus generates a diverse array of natural products, including macrolides, tetracyclines, aminoglycosides, glycopeptides, ansamycins, and terpenes, all of which exhibit significant structural diversity [14,15].

Initial screening and discovery of novel lead molecules typically start with crude extract screening, followed by bioactivity-guided fractionation and purification [16]. However, this method faces challenges due to the complexity of the samples, which can lead to additive or multiplicative effects from various compounds present in the extract, as well as potential rediscovery of already known compounds or their analogs. This issue can be partially alleviated by pre-fractionating crude extracts and employing advanced dereplication strategies using fast and highly accurate methods, such as high-performance liquid chromatography coupled with high-resolution tandem mass spectrometry (HPLC-HRMS/MS) to develop high-throughput discovery pipelines [17,18,19,20].

Another challenge involves cryptic genes that can hinder the production and detection of desired bioactive lead compounds using conventional methods. These cryptic biosynthetic gene clusters (BGCs) can be identified through genome sequencing. According to genomics analysis, many bacteria with large genomes possess the coding capacity to produce various secondary metabolites, including antibiotics, antitumor agents, and immunomodulatory drugs [21,22]. The growing accessibility of sequencing and assembling microbial genomes allows for the exploration of the biosynthetic potential of organisms that produce valuable secondary metabolites. This can also be combined with metabolomics, which identifies the secondary metabolites produced. Therefore, integrating metabolomics with genomics represents a new trend in studying the chemistry of natural products [21,23].

In our ongoing efforts to isolate new bioactive molecules [24], it is essential to explore new groups of actinomycetes from untapped environments or under-exploited conditions as potential sources of novel bioactive secondary metabolites. In this study, we integrated genomics and metabolomics to study *Streptomyces tunisiensis* GCF 039538125, which was previously isolated from a nudibranch collected in a coastal region of the Red Sea, Egypt [10]. While many natural products have been isolated from nudibranchs, their associated microbial communities and microbial natural products are understudied compared to those of other organisms, and therefore, may present an opportunity to discover new natural products. Ultra-high-performance liquid chromatography–high-resolution mass spectrometry (UHPLC-HRMS/MS) and the Global Natural Products Social Molecular Networking Project (GNPS) were used to gain a comprehensive understanding of the whole metabolic profile of this isolate. In addition, whole-genome sequencing with Illumina MiSeq was used to identify the BGCs responsible for producing these metabolites.

## 2. Results and Discussion

### 2.1. Whole-Genome Sequencing and Genomic Analysis

Preliminary 16S rRNA sequencing and morphological characteristics of strain 34 indicated that the microorganism belongs to the genus *Streptomyces* [10]. The whole-genome sequencing of *Streptomyces* sp. was accomplished by using Illumina MiSeq and genome assembly by MIGA online to generate a draft genome of 7,313,906 bp, with 96% completeness and with G+C content of 72.09%. The genome was assembled into 964 contigs with N50s of 12,826 bp. This level of fragmentation is typical for assemblies based solely on Illumina short reads and may affect the continuity of some BGCs. The sequence was deposited in the NCBI database (accession number PRJNA1251220 and BioSample SAMN48000432). The closest genome to strain 34 was *Streptomyces tunisiensis GCF 039538125 1* (accession number JCM 17589) with an identity of 99.11%, OrthoANIu value of 99.23%, DDH estimate (GLM-based) of 93.60% [91.8–95.1%], and the distance between the two being 0.0082. The alignment of this isolate genome against those of *Streptomyces tunisiensis* using MIGA annotation revealed similar predicted protein content and functions between the two organisms, with an amino acid identity of 99.2%, and 7191 protein-coding genes. *S. tunisiensis* is an actinomycete bacterium originally isolated from northern Tunisian soil [25], with a marine strain later identified in Abu-Qir sediments, Egypt, demonstrating its environmental adaptability. This species exhibits potent antibacterial activity, particularly against methicillin-resistant *Staphylococcus* species and other Gram-positive and Gram-negative bacteria. Furthermore, the marine strain produces a bioactive pigment with antimicrobial, antiviral, antioxidant, and antitumor properties, underscoring its promising potential for medical and pharmaceutical applications [26]. Moreover, it was reported that *S. tunisiensis* DSM 42037 produces significant amounts of free ferulic acid, demonstrating its ability to secrete feruloyl esterase. This highlights its potential as a prolific and safe source of bioactive secondary metabolites for agricultural and pharmaceutical uses, in addition to antimicrobial and antitumor activity [27].

### 2.2. Secondary Metabolite Profiling and Genome Mining

The potential of this *Streptomyces* strain to produce secondary metabolites was analyzed using Antibiotics & Secondary Metabolite Analysis Shell (antiSMASH v5.1.2), which showed that it contains numerous BGCs, including polyketide, non-ribosomal peptides, terpenes, aminoglycosides, and RiPPs in the bacterial genome. AntiSMASH predicted 36 putative secondary metabolite clusters. The predicted clusters of secondary metabolites, along with their ecological distribution, the microorganisms harboring these clusters, and their bioactivity, are shown in Table S1. The results showed that this strain contains 15 gene clusters for synthesizing peptides and polyketides. Most of these clusters were NRPSs and shared no similarity to characterized NRPSs with known natural products, suggesting that these pathways may encode new natural products. This *Streptomyces* sp. has eight BGCs with 100% similarity to known clusters, including two RiPPs, T2PKS, ecotine, siderophore, lassopeptide, and terpene. It contains eight NRPS biosynthetic gene clusters, six of which had a unique non-ribosomal peptide prediction, which is interesting due to non-matching identity or its low similarity with other known BGCs. Only two clusters show 81 and 12% similarity to antimycin, suggesting that this *S. tunisiensis* isolate may encode new chemical diversity. To further characterize the unidentified lanthipeptide, its BGCs genome was analyzed with BAGEL [28], which specializes in the detection and annotation of RiPPs such as lanthipeptides. BAGEL predicted the same Lanthipeptide as antiSMASH without providing any information for unidentified Lanthipeptides. In addition to antiSMASH, DeepBGC, another genome mining tool for BGC identification, was used. DeepBGC detected 49 BGCs in the assembled contigs, and about 84% of these BGCs were predicted to produce metabolites with bioactivity; the majority (30 BGCs) were predicted to have antimicrobial activity, while 1 BGC was predicted to produce a cytotoxic metabolite (Table S2).

**Figure 1:**
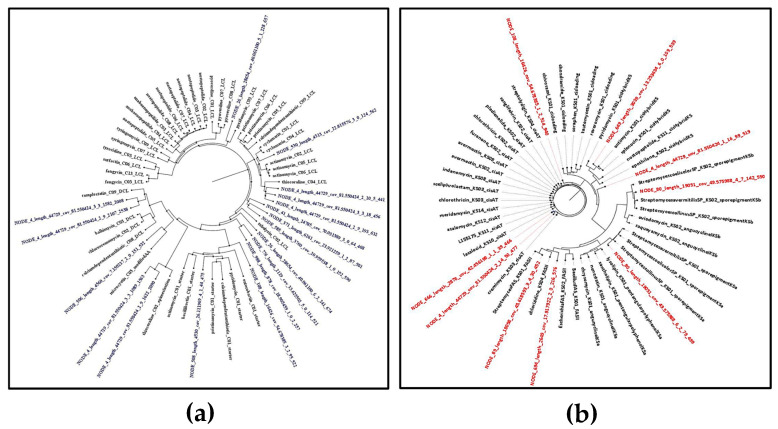
Maximum likelihood phylogenetic tree of non-ribosomal peptide (C domains) and polyketides (KS domain) detected by NAPDoS against the NaPDoS domain database: (**a**) C domains—domains from this *Streptomyces* isolate are colored blue; (**b**) KS domains—domains from this *Streptomyces* isolate are colored red.

NaPDoS 2 was used to analyze the C and KS domains of the detected BGCs in the assembled contigs. For NRPSs, 18 preliminary C domain candidates were detected. The phylogenetic analysis (Figure 1a) of these domains with the closest reference NaPDoS database matches revealed that some of these domains were found to be closely related to the C domains of some bioactive compounds BGCs such as the anticancer thiocoraline [29], the antibiotic pyridomycin, which has potent activity against mycobacteria and some Gram-negative bacteria [30], and the antimycobacterial and antiplasmodial cyclopeptides cyclomarines [31].

NaPDoS could also detect 9 preliminary KS domain candidates. Some of these KS domains were also found to be phylogenetically closely related to biosynthetic genes that prescribe the biosynthesis of some bioactive compounds, such as the cytotoxic chlorizidine [32] and the antibiotics cremimycin [33], antimycin, and pyridomycin [30] (Figure 1b). However, some of the detected KS and C domains showed low sequence identity scores to their predicted natural product, as shown in Tables S3 and S4. These results suggest potential novelty in the NRPS and PKS products. Analysis of genomic data revealed that the majority of gene clusters were related to biosynthetic gene clusters that produced metabolites with demonstrated antimicrobial and cytotoxic bioactivity; this supports our prior findings that this strain possesses desirable antimicrobial and antitumor properties, encouraging the exploration of this *S. tunisiensis* strain’s metabolic profile to gain deeper insights into its natural products. However, such similarity in C and KS domains does not directly predict the activity of the resulting metabolites, but rather indicates possible biosynthetic relatedness.

### 2.3. Metabolomics Analysis

**Figure 2:**
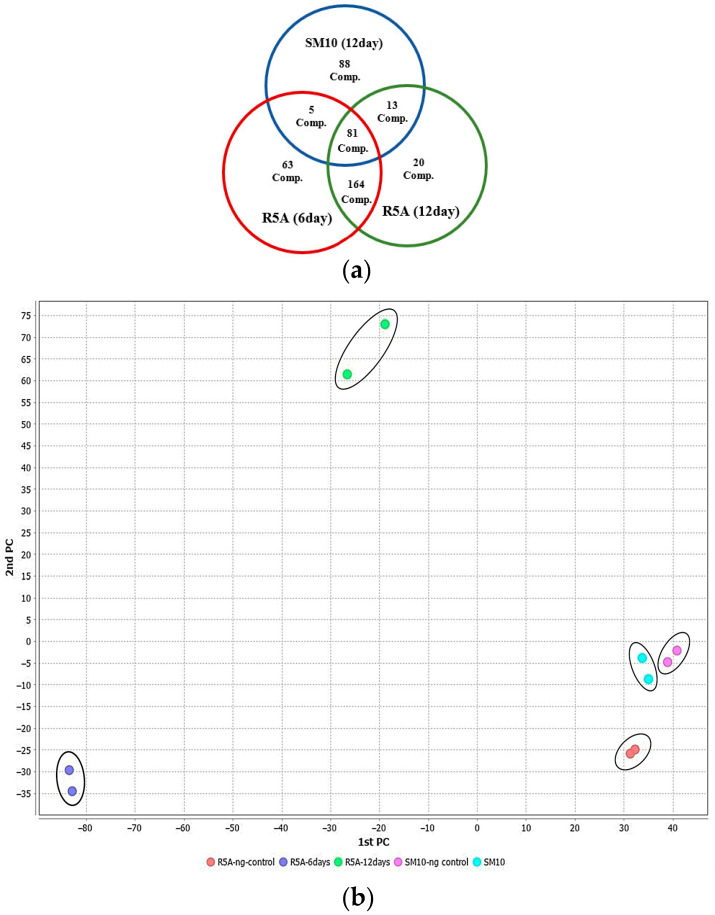
Metabolites of the *S. tunisiensis* isolate: (**a**) unique and common metabolites after growth in R5A and SM10 media; (**b**) two-dimensional plot of metabolites produced in SM10 and R5A media by PCA.

The metabolic profile of this *S. tunisiensis* strain isolated from *C. quadricolor* was investigated by conducting LC-MS/MS dereplication on extracts from the combined culture pellet and supernatant in two different media, SM10 and R5A, over periods of 6 and 12 days. The analysis of LC-MS/MS chromatograms revealed that the production of secondary metabolites by this *S. tunisiensis* strain varies significantly between the two media. By processing the MS/MS data obtained from positive ion mode, correcting for charge, removing adducts, and subtracting background and media signals, a molecular network was constructed using GNPS [34]. This network encompassed 738 nodes, with parent mass molecular sizes ranging from 177 to 1792 Da (Figure S1). Notable differences in metabolite profiles emerged between the two-culture media. By comparing the spectra against public databases, a total of 86 distinct compounds were identified. In total, 569 metabolites featuring unique parent masses and fragmentation patterns were detected. The greater production of bacterial metabolites in the R5A medium, compared to the SM10 medium, is likely attributable to the richer carbon sources present in R5A. Clear differentiation in metabolomic profiles between R5A and SM10 indicates variations in the metabolism of the *S. tunisiensis* strain depending on the medium used (Figure 2a). Principal component analysis (PCA) plots were generated to visualize the metabolomic profiles of samples collected at 6 and 12 days from both media types (Figure 2b). The PCA indicated clear differences in bacterial metabolite patterns between the two media, with replicates clustering closely within each group. A distinct separation was observed between the R5A and SM10 profiles, highlighting that the metabolic activity of the Streptomyces isolate varied significantly based on the growth medium used. Overall, the metabolite profiles demonstrated a rich diversity of natural products. The analysis revealed a wide range of natural products, including fatty acids, polyketides, proteins, terpenoids, alkaloids, and flavonoids. Some identified metabolites included cholesterol, desferrioxamines, isomyristic acid, lumichrome, kaurane, bisucaberin, barceloneic acid A, and ceratodictyol.

**Figure 3:**
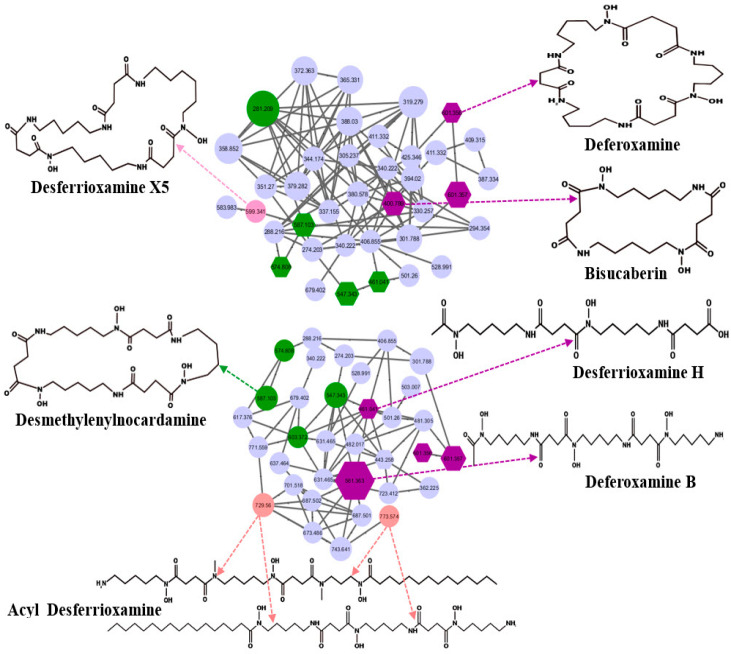
Desferrioxamine subnetwork by GNPS presented by the node and the edge graph produced by *S. tunisiensis*: purple nodes represent potential deferoxamine and bisucaberin; pink and green nodes represent desferrioxamine derivatives that were annotated using MS/MS data; GNPS cosine cutoff, 0.7.

GNPS identified desferrioxamines from UHPLC-HRMS data. The ions corresponding to desferrioxamine H ([M+H]^+^, *m*/*z* 461.267) and desferrioxamine E ([M+Na]^+^, *m*/*z* 623.337) were detected (Figure 3 and Table S5). Bisucaberin, a dihydroxamate siderophore, cyclic peptide ([M+H]^+^, *m*/*z* 401.251), was also discovered in the *Streptomyces* extract in SM10 medium. Bisucaberin previously isolated from *Alteromonas haloplanktis* strain SB-1123 was shown to have direct cytotoxicity on tumor cells [35]. Moreover, Desferrioxamine X5 ([M+H], *m*/*z* 599.3409) has been reported in *Streptomyces olivaceus* [36,37]. Ferrioxamine D1+Al (M-2H+Al, *m*/*z* 627.3292) was previously isolated from *Streptomyces* sp. [38].

The GNPS-identified terpenoid kaurane ([M+H]^+^, *m*/*z* 273.29) is an active tetracyclic diterpenoid involved in both primary and secondary metabolism of plants, possessing antimicrobial and cytotoxic activity with apoptosis-inducing action [39,40].

### 2.4. Correlating the Metabolome and the Genome

**Figure 4:**
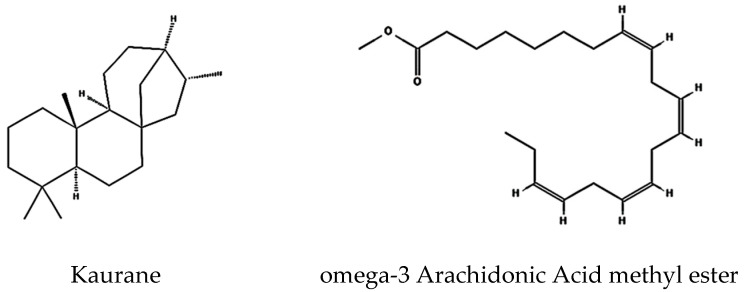
Identified compounds produced by *S. tunisiensis* by GNPS.

Integrating metabolomic and genomic analyses offers a comprehensive understanding of bioactive compounds biosynthesis, and identifying their BGCs is essential. In some instances, a BGC has been identified and linked to a known compound, yet the corresponding metabolite was not detected in the extracts. For example, antiSMASH identified a lanthipeptide and another gene cluster with 100% similarity to venezuelin and curamycin in the genome. Still, neither compound was present in the analyzed culture extract. Conversely, several metabolites identified through UHPLC-HRMS and GNPS analysis could not be associated with BGCs detected by antiSMASH. For example, GNPS spectral analysis identified diterpene, terpenoid, and polyketide products. The spectra closely matched reference spectra for kaurane (Figure 4) from bronze datasets in the GNPS database, with very low mass error. While diterpene and polyketide were detected, no corresponding terpene or polyketide synthase-encoding genes were identified in the genome. One possible explanation for this mismatch is the limitation of short-read sequencing. The genome assembly in this study was generated exclusively from Illumina MiSeq paired-end reads, which, while highly accurate, may result in fragmented assemblies and incomplete representation of biosynthetic gene clusters (BGCs). Incorporating long-read sequencing technologies (e.g., PacBio, Oxford Nanopore) or hybrid assembly approaches in future work would improve contiguity and allow for more comprehensive BGC reconstruction. Moreover, the low genome–metabolome match rate might be due to suboptimal culture conditions or silent BGCs. Some of the gene clusters are only expressed under certain stress conditions, which were not simulated in the cultures. Some others are currently unknown and are yet to be identified and added to the database.

Among the metabolomic and genomic data, only two molecules, a siderophore and a lassopeptide, were detected in both genomic data with 100% similarity to known BGCs and identified in the metabolic extracts. Notably, the detected siderophores and the promising peptide exhibited high abundance and intense UV absorbance.

The BGCs of desferrioxamines identified in the *S. tunisiensis* strain have 100% similarity with BGCs from known desferrioxamine producers such as *Streptomyces griseus* and *Streptomyces* sp. ID38640 [41]. The siderophore (ferrioxamine H) was also detected in the *S. tunisiensis* isolate extract ([M+H]^+^, *m*/*z* 461.261), but we were unable to identify an associated BGC in this organism as possibly responsible for production of ferrioxamine H. However, antiSMASH analysis showed that this *S. tunisiensis* isolate contains additional siderophore BGCs of unknown function (Table S1). Since the siderophores are produced by many Actinomycetes and are involved in cellular growth and survival [42,43], detecting them in cell extract is not surprising.

**Figure 5:**
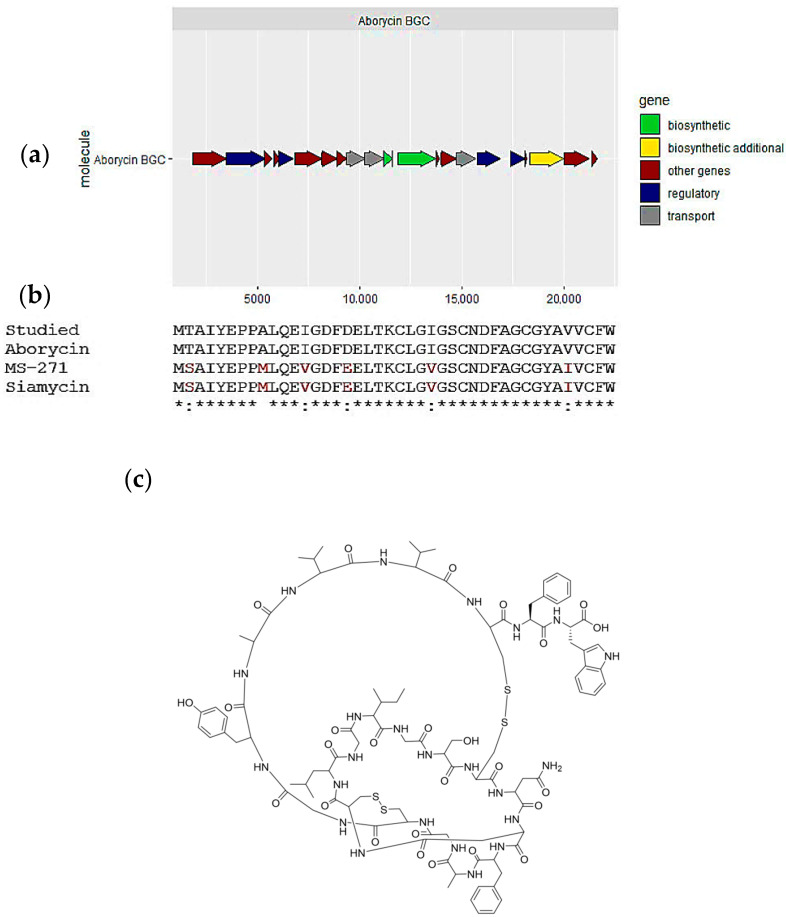
Aborycin, one of the secondary metabolites predicted in the *Streptomyces tunisiensis* isolate: (**a**) biosynthetic gene cluster for aborycin; (**b**) alignment of the predicted lassopeptide structure and most similar cluster products, aborycin and siamycin and MS-271; and (**c**) Aborycin chemical structure.

The BGC responsible for the lassopeptide was identified in the *S. tunisiensis* isolate. This gene cluster exhibited 100% similarity to three known clusters: Aborycin, MS-271, and Siamycin (see Figure 5a). Moreover, a compound was detected in the extract as ([M+H]^2+^, [M+3H]^3+^, *m*/*z* 2162.855) and identified as siamycin based on GNPS derived from UHPLC-HRMS data. The predicted peptides generated with antiSMASH were aligned with the three peptides produced with known clusters (see Figure 5b and Table S6). We found that the peptide identity is 100% with aborycin (Figure 5c), while 90.5% identity with MS-271 and Siamycin. This is due to a substitution for isoleucine and valine in the core peptide and the difference in some amino acids in the leader peptide.

**Figure 6:**
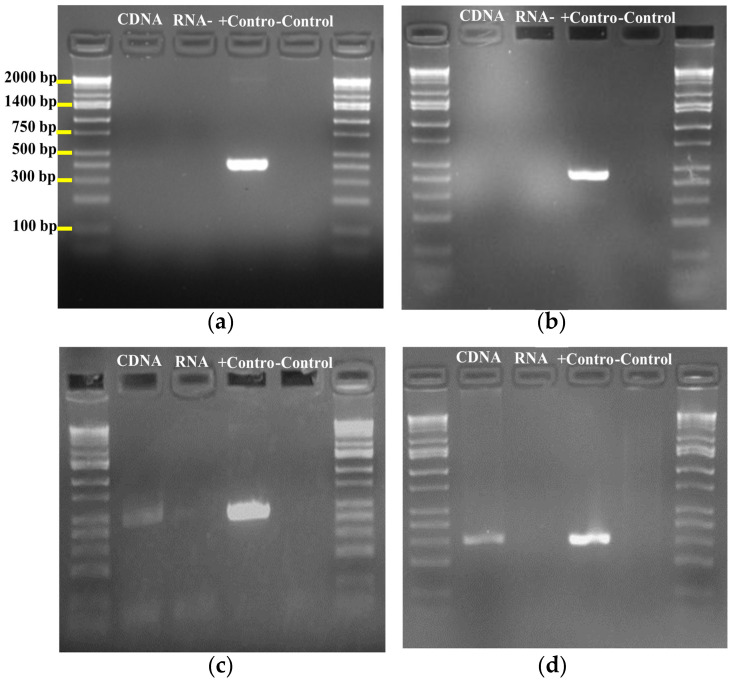
PCR amplicons of RIPPs from genomic DNA and cDNA of *S. tunisiensis* strain (**a**) Lanthipeptide node 6 size 389 bp; (**b**) Lanthipeptide node 71 size 440 bp; (**c**) lasso peptide node 54 amplicon size 459 bp; (**d**) Lassopeptide node 54 amplicon size 298 bp.

To correlate the metabolome with the genome, large-scale cultures were conducted to produce significant amounts of the peptide; the molecule was isolated using a semi-preparative method, and ^1^H, ^13^C and 2D COSY and HSQC NMR spectra (Figures S2–S5) confirmed that it is a RiPP. Furthermore, to validate that the identified gene cluster is responsible for this peptide, we designed six degenerate primers to determine if the RiPPs genes are expressed in cultures of the *S. tunisiensis* isolate. We identified three RiPPs: one lassopeptide, coded as Node 54, and two lanthipeptides, coded as Node 6 and Node 71. Notably, only the lassopeptide showed positive cDNA PCR amplification for the core biosynthetic gene (Figure 6), indicating that this lassopeptide is produced during culture conditions.

*Streptomyces griseoflavus* produces the aborycin lassopeptide, and the associated BGC has been identified in *Streptomyces* sp. scsio ZS0098 isolated from the deep sea [44]. In the current study, aborycin was detected in the extract as ([M+H]^2+^, [M+3H]^3+^, *m*/*z* 2162.855). The same *m*/*z* 2162.8541 was previously detected in *Streptomyces* sp. scsio ZS0098 [44]. LC-MS analysis of the culture extract of the *S. tunisiensis* isolate indicated the presence of lassopeptide compared to aborycin detected in *Steptomyces coelicolor* (Figure S6). The isotopic distribution of the studied molecules and aborycin is the same. Therefore, our genomics and metabolomics results indicate that this *Streptomyces* strain. isolated from the Red Sea nudibranch produces the same molecule as other *Streptomyces* isolated from the deep sea. Our findings support the hypothesis that marine actinomycetes can produce novel bioactive molecules and highlight the value of nudibranchs for future metagenomic studies, especially BGCs. We evaluated a gene cluster that was not expressed in culture using deep pyrosequencing of *S. tunisiensis*, which could help optimize growth techniques to enhance and/or induce the expression of these valuable BGCs producing promising natural products. We identified a gene cluster encoding a lanthipeptide, aborycin, using deep pyrosequencing of the *S. tunisiensis* strain that was not expressed in culture conditions (cDNA and metabolite analysis). Optimization of growth techniques and conditions may result in the enhancement or induction of the BGC, leading to the production of novel natural products.

## 3. Materials and Methods

### 3.1. Streptomyces Cultivation and Whole-Genome Sequencing

For isolate cultivation, a single colony was inoculated into 5 mL of R2A broth and incubated at 30 °C for 5 days with shaking at 150 rpm. The DNA was extracted from the cultivated isolate according to the manufacturer’s instructions for actinomycetes of the Quick-DNA Fungal/Bacterial Miniprep Kit (Zymo Research, Irvine, CA, USA), with some modifications as described in our previous study [10]. The integrity of the isolated DNA was assessed using agarose gel electrophoresis and the DNA was stored at −20 °C.

Preliminary isolate identification was performed by PCR amplification and sequencing of the 16S rDNA gene using primers 27F and 1412R as described previously [10]. Subsequently, the genome was sequenced using Illumina MiSeq (Illumina, Inc., San Diego, CA, USA), in paired-end format with a read length of 300 bp. The DNA library was constructed using the Nextera XT DNA sample prep kit (Illumina), verified on the 2100 Bioanalyzer (Agilent, Santa Clara, CA, USA), and sequenced on an Illumina MiSeq instrument using a 600-cycle paired-end MiSeq reagent V3 kit with 5% PhiX.

### 3.2. Bioinformatics Analysis and Ecological Statistics

DNA Sequences were trimmed and quality filtered using Trim Galore version 0.6.4 (Phred score ≥ 25; minimum sequence length, 100 nucleotides) [45], and assembled using default settings in SPAdes v3.13.0 [46]. The assembly was then verified using Quast 5.0.2 [47], and genome completeness was calculated using MiGA online [48]. For phylogenetic analyses, the sequences were aligned using DFAST_QC and MiGA [48,49]. The taxonomic identity was checked using ANI calculate of EzBiocloud [50] and Type Strain Genome Server (TYGS) [51] to calculate ANI, dDDH and distance between the genome of the studied isolate and most related reference genome. Contigs were then annotated using Prokka 1.14.0 [52]. The secondary metabolite BGCs were predicted and identified using antiSMASH v5.1.2, DeepBCG 0.1.31, and NaPDoS2 [53].

### 3.3. UHPLC HRMS Analysis

To generate the secondary metabolic profile of the isolated *Streptomyces* sp., the metabolic extract was prepared as described in our earlier study [24]. Briefly, 500 μL of the starter culture was transferred into duplicate Erlenmeyer flasks containing 50 mL of medium and incubated at 30 °C with shaking at 150 rpm for 12 days. Subsequently, 25 mL of culture and corresponding negative controls were harvested after 6 and 12 days from R5A medium, and after 12 days from SM10 medium (owing to limited growth within the first 6 days) and then centrifuged, in order to examine secondary metabolite production profiles. The metabolic extracts from both the filtrate and the pellet were analyzed to detect a wide range of secondary metabolites. The extracts were examined using LC-MS/MS with an Agilent 1290 Infinity II UHPLC system (Agilent Technologies, Waldbronn, Germany) coupled to a Bruker Impact II ultra-high resolution Qq-TOF mass spectrometer (Bruker Daltonics, GmbH, Bremen, Germany) equipped with an ESI source, as described in our previous study, A Kinetex™ (Phenomenex, Torrance, CA, USA) 1.7 µm UHPLC (C18) column (50 × 2.1 mm) was used for chromatographic separation. MS spectra were acquired in positive ionization mode from *m*/*z* 50–2000 Da. Metabolic extracts of the Streptomyces strain were resuspended in 1 mL methanol (LC-MS grade) and directly analyzed. After injection with 10 µL of the metabolic extract, it was separated using a gradient of water (A) and acetonitrile 100% (B) with 0.1% formic acid and at a flow rate of 0.5 mL/min throughout the run. The gradient elution was initiated at 5% solvent B for 3 min and then at a linear gradient of 5% to 50% B over 5 min and held at 50% B for 2 min followed by a linear gradient of 50% to 100% B over 5 min and held at 100% B for 3 min. The column was then re-equilibrated to 5% B for 1 min [24].

### 3.4. Molecular Networking and Spectrum Annotation

The mass spectrometry data were converted to mzXML format using MS-Convert software version 3.0 (ProteoWizard, CA, USA). Subsequently, the data were dereplicated using GNPS Networking. The online GNPS workflow was employed to create a molecular network in positive ion mode. The MS2 raw data were clustered with MS-Cluster with a parent mass tolerance of 0.02 Da and a MS2 fragment ion tolerance of 0.05 Da to create consensus spectra. The edges were filtered to have a cosine score above 0.7 and more than 4 MS2 matched peaks were used. Edges between nodes were preserved if both nodes were within each other’s top 10 most similar nodes. GNPS output can be found through this link, https://gnps.ucsd.edu/ProteoSAFe/static/gnps-splash.jsp?redirect=auth (accessed on 1 September 2025). Due to the lack of hints when using the strict cutoff window, and to explore other possibilities, we performed the chemical analysis [34]. To determine the clustering of metabolites within different media, principal component analysis (PCA) was generated using MZmine 2.53. Raw data files were processed using MZmine2 for feature detection [54]. MS1 spectra were filtered with a noise threshold of 10,000, while MS2 spectra were filtered at a threshold of 500. The filtered data underwent several processing steps with the following parameters: chromatogram builder (minimum time span: 0.03 min; minimum intensity of the highest data point: 1500; *m*/*z* tolerance: 5 ppm), chromatogram deconvolution using the local minimum search algorithm (MS2 scan pairing within 0.030 Da *m*/*z* range and 0.2 min retention time window), isotopic peak grouper (*m*/*z* tolerance: 5 ppm; retention time tolerance: 0.1 min; maximum charge: 3; representative isotope: lowest), join aligner (*m*/*z* tolerance: 5 ppm; retention time tolerance: 0.01 min), feature list row filter (*m*/*z* range: 200–2000 Da; MS/MS filter applied), and peak finder (intensity tolerance: 0.1; retention time tolerance: 0.1 min; *m*/*z* tolerance: 5 ppm). The resulting molecular network was visualized using Cytoscape version 3.7.1 [55]. The generated mass spectrometry data have been deposited in the MassIVE repository under the MassIVE ID# MSV000097640.

### 3.5. Large-Scale Bacterial Metabolite Extraction

*Streptomyces* sp. was cultivated in R5A medium. A 5 mL portion of the culture was used to inoculate six 2.5 L Erlenmeyer flasks, each containing 500 mL of R5A. The flasks were incubated at 30 °C while shaking at 150 rpm for 12 days. After incubation, the cultures were collected and centrifuged at 3500 rpm for 25 min. Secondary metabolites were detected in both the filtrate and the cell pellet extracts. The metabolites from the filtrate were extracted using an equal volume of ethyl acetate, with the extraction process conducted at 150 rpm for 1 h at room temperature, repeated three times. The cell pellets were frozen at −80 °C for 1 h. Following this, 300 mL of methanol was added, and the mixture was sonicated for 20 min, then centrifuged at 3500 rpm for 15 min. This extraction process was also repeated three times. The methanolic extract of the pellet was then combined with the filtrate extract. The combined extract was dried using a speed vacuum, lyophilized, and subsequently analyzed directly by LC-MS/MS.

#### 3.5.1. Semi-Preparative UHPLC Sample Preparation of Molecules of Interest

The *Streptomyces* metabolic extract was processed using chromatography on a Luna C18 reversed-phase LC column (5 μm, 250 mm × 10 mm). The chromatography solvents used were solvent A (water with 0.01% (*v*/*v*) trifluoroacetic acid (TFA)) and solvent B (acetonitrile (ACN) with 0.01% (*v*/*v*) TFA). The flow rate was maintained at 2 mL/min throughout the process. The elution profile employed for isolating the target compound included: 5% solvent B for 5 min, a linear gradient from 5% to 100% solvent B over 60 min, a hold at 100% solvent B for 3 min, a return to 5% solvent B over 1 min, 5% solvent B for 1 min, a quick gradient from 5% to 100% solvent B over 1 min, 100% solvent B for 3 min, a transition back to 5% solvent B over 1 min, and 5% solvent B for an additional 2 min.

#### 3.5.2. Purification and NMR Analysis of Molecules of Interest

An extra isocratic purification step was performed using 48% solvent B for 50 min. The solvent was then removed under vacuum to yield the dried compound. The final yield was 2 mg/L media. The dried powder was dissolved in 500 µL of DMSO-d6, sonicated for 3 min, and transferred into a standard 5-mm NMR tube. NMR spectra, including ^1^H, ^13^C, COSY, and HSQC, were recorded on a Bruker Advance III HD (700 MHz) equipped with a 5 mm indirect broadband cryoprobe. Data processing and analysis were conducted using Topspin 3.5 (Bruker, Karlsruhe, Germany).

### 3.6. Screening for Ribosomally Synthesized and Post-Translationally Modified Peptides (RiPPs) Gene Clusters Expression by PCR

To detect the BGC responsible for producing the molecule of interest, the *Streptomyces* isolate was screened for expressed RiPP gene clusters. Degenerate primers were designed using Geneious Prime 2020.0.4 (USA) based on conserved motifs within the core biosynthetic regions of known RiPP gene clusters, ensuring broad yet specific detection across diverse genomes. The total RNA of *Streptomyces* isolate was extracted using the *Quick*-RNA^TM^ Miniprep Kit (Zymo Research, Irvine, CA, USA) according to the manufacturer’s instructions and then treated with DNase I (New England BioLabs, Ipswich, MA, USA), followed by purification using RNA Clean & Concentrator^TM^-5 (Zymo Research, Irvine, CA, USA). Subsequently, the cDNA was generated using ProtoScript^®^ First Strand cDNA Synthesis Kit (New England BioLabs, Ipswich, MA, USA) according to the manufacturer’s instructions. Six sets of degenerate primers were designed (Table 1) to amplify the core biosynthetic genes for three RiPPs detected by antiSMASH, via PCR using 50 ng/µL of the cDNA as a template. The expression of RiPPs gene clusters was assessed by amplification of the core biosynthetic gene involved in peptide biosynthesis. All PCRs were performed in a 20 µL PCR with Phire Green Hot Start II DNA Polymerase (Fisher Scientific, Waltham, MA, USA). Each PCR contained Phire Green Reaction Buffer, 10 mM dNTPs (New England Biolabs, Ipswich, MA, USA), 20 mg/mL DMSO, 10 pmol of each primer, and 1 μL Phire Hot Start II DNA Polymerase. The thermocycling conditions were as follows: initial denaturation was performed at 98 °C for 30 s, followed by 35 cycles of denaturation at 98 °C for 5 s, annealing Tm and time depending on the primer sequence, extension at 72 °C for 5 s, with a final extension at 72 °C for 1 min. Genomic DNA was used as a positive control, and two negative controls were used: negative cDNA and water. The resulting amplicons were visualized by agarose gel electrophoresis in 1% agarose.

## 4. Conclusions

Marine actinomycetes are a wealth of diverse natural products and are a potential source of novel bioactive molecules. In this study, whole-genome sequencing of a *Streptomyces tunisiensis* strain associated with a nudibranch was conducted to screen for the presence of natural product BGCs. We found through secondary metabolite profiling and genome mining that this *S. tunisiensis* strain contains numerous BGCs, including polyketide, non-ribosomal peptides, terpenes, aminoglycosides, and RiPPs. Thirty-six putative secondary metabolite clusters were predicted by antiSMASH, with only eight known clusters, suggesting that these pathways may encode new natural products. The metabolic profile of this *S. tunisiensis* strain uncovered a wide range of natural products, including fatty acids, polyketides, proteins, terpenoids, alkaloids, and flavonoids. Interestingly, the lassopeptide aborycin was identified and confirmed in the metabolomic and genomic data. We identified several compounds based on standards and public spectral libraries, and antiSMASH suggests that the *S. tunisiensis* strain harbors several novel bioactive gene clusters that could biosynthesize compounds that may protect nudibranchs from infection and predators. This study also highlights the importance of integrating metabolomic and genomic analyses to understand bioactive compounds and their biosynthesis comprehensively.

## Figures and Tables

**Table 1:** List of designed primers used in this study.

Target Gene	Primer	Sequences	Approximate Amplicon Length (bp)	References
Node 6	577F	CTCTGGTCGCCCTCTTGAAG	389	This study
	966R	GCAGGTCGAGACTGATCCTG		
	128F	CATGACCTCACAGCCCATCA	227	
	355R	CCGCTGATCAGGTCGTACTC		
Node 54	2F	GTCATCCCTCATGGCATATG	298	This study
	300R	GTCGACGGAACACCAGAAG		
	90F	CATCGCTGAACTGTGTGACG	459	
	549R	CAATCCGGAACTGTTCTGCC		
Node 71	287F	GGTTCTCCATGGCTGTCTCC	440	This study
	727R	GAATCGGTTCCTTCCTGCTC		
	1615F	GCGGGATCCTCACTCATCAG	190	
	1805R	GACTACTACAGCCTGGGTGC		

## Data Availability

All data generated or analyzed during this study are included in this published article and its Supplementary Information files.

## Supplementary Materials

The following supporting information can be downloaded at: https://www.mdpi.com/article/10.3390/md23100404/s1, Table S1: Summary of the antiSMASH biosynthesis cluster prediction for the Streptomyces sp; Table S2: Summary of the DeepBGC biosynthesis cluster prediction for the Streptomyces sp; Table S3: Summary of the napdos biosynthesis cluster prediction KS domain for the Streptomyces sp; Table S4: Summary of the napdos biosynthesis cluster prediction C domain for the Streptomyces sp; Table S5: Spectral information (LC-MS/MS) for compounds identified through GNPS. For all compounds: Ion source: LC-ESI; MS-Level: MS2; Instrument; qTOF; Ionization mode: Positive; MS Category: Experimental; Table S6: The sequence alignment of the leader/core regions within the cluster; Figure S1. Molecular networking of metabolites produced by S. tunisiensis generated by Cytoscape version 3.7.1. (GNPS cosine cutoff 0.7); Figure S2. ^1^H NMR spectra for the isolated molecule (Aborycin); Figure S3. 13C NMR spectra for the isolated molecule (Aborycin); Figure S4. COSY NMR spectra for the isolated molecule (Aborycin); Figure S5. HSQC NMR spectra for isolated molecule (Aborycin); Figure S6. LC-MS comparative analysis of lassopeptide; (a) studied molecules accumulated in the culture of Streptomyces tunisiensis. (positive mode) and (b) aborycin accumulated in culture of Streptomyces coelicolor. Further inquiries can be directed to the corresponding author.

## Abbreviations

The following abbreviations are used in this manuscript:

BGCs	Biosynthetic gene clusters.
GNPS	Global natural products social molecular networking project.
HPLC-HRMS/MS	High-resolution tandem mass spectrometry.
MS	Mass *spectrometry*.
NRPS	Non-ribosomal peptide synthetase.
PKS	Polyketide synthase.
RiPPs	Ribosomally synthesized and post-translationally modified peptides.
UHPLC-HRMS/MS	Ultra-high-performance liquid chromatography–high-resolution mass *spectrometry*.

## References

[1] Teta R., Marteinsson V.T., Longeon A., Klonowski A.M., Groben R., Bourguet-Kondracki M.-L., Costantino V., Mangoni A. (2017). Thermoactinoamide A, an Antibiotic Lipophilic Cyclopeptide from the Icelandic Thermophilic Bacterium *Thermoactinomyces vulgaris*. J. Nat. Prod..

[2] Atanasov A.G., Zotchev S.B., Dirsch V.M., Orhan I.E., Banach M., Rollinger J.M., Barreca D., Weckwerth W., Bauer R., Bayer E.A. (2021). Natural products in drug discovery: Advances and opportunities. Nat. Rev. Drug Discov..

[3] Abbas A., Barkhouse A., Hackenberger D., Wright G.D. (2024). Antibiotic resistance: A key microbial survival mechanism that threatens public health. Cell Host Microbe.

[4] Ahmed S.K., Hussein S., Qurbani K., Ibrahim R.H., Fareeq A., Mahmood K.A., Mohamed M.G. (2024). Antimicrobial resistance: Impacts, challenges, and future prospects. J. Med. Surg. Public Health.

[5] Ahmed I., Asgher M., Sher F., Hussain S.M., Nazish N., Joshi N., Sharma A., Parra-Saldívar R., Bilal M., Iqbal H.M.N. (2022). Exploring Marine as a Rich Source of Bioactive Peptides: Challenges and Opportunities from Marine Pharmacology. Mar. Drugs.

[6] Blunt J.W., Carroll A.R., Copp B.R., Davis R.A., Keyzers R.A., Prinsep M.R. (2018). Marine natural products. Nat. Prod. Rep..

[7] Wahidullah S., Guo Y.W., Fakhr I.M., Mollo E. (2006). Chemical diversity in *opisthobranch* molluscs from scarcely investigated Indo-Pacific areas. Prog. Mol. Subcell Biol..

[8] Dean L.J., Prinsep M.R. (2017). The chemistry and chemical ecology of nudibranchs. Nat. Prod. Rep..

[9] Karuso P., Scheuer P.J. (1987). Bioorganic Marine Chemistry, Berlin, Heidelberg, 1987. Chemical Ecology of the Nudibranchs.

[10] Abdelrahman S.M., Patin N.V., Hanora A., Aboseidah A., Desoky S., Desoky S.G., Stewart F.J., Lopanik N.B. (2021). The natural product biosynthetic potential of Red Sea nudibranch microbiomes. PeerJ.

[11] Nguyen C.T., Dhakal D., Pham V.T.T., Nguyen H.T., Sohng J.-K. (2020). Recent Advances in Strategies for Activation and Discovery/Characterization of Cryptic Biosynthetic Gene Clusters in *Streptomyces*. Microorganisms.

[12] Sharma V., Kaur R., Salwan R. (2021). *Streptomyces*: Host for refactoring of diverse bioactive secondary metabolites. 3 Biotech.

[13] Sarmiento-Tovar A.A., Silva L., Sánchez-Suárez J., Diaz L. (2022). *Streptomyces*-Derived Bioactive Pigments: Ecofriendly Source of Bioactive Compounds. Coatings.

[14] Alam K., Mazumder A., Sikdar S., Zhao Y.M., Hao J., Song C., Wang Y., Sarkar R., Islam S., Zhang Y. (2022). *Streptomyces*: The biofactory of secondary metabolites. Front. Microbiol..

[15] Harir M., Bendif H., Bellahcene M., Fortas Z., Pogni R., Enany S. (2018). Streptomyces Secondary Metabolites. Basic Biology and Applications of Actinobacteria.

[16] Klebe G., Klebe G. (2024). Screening Technologies for Lead Structure Discovery. Drug Design: From Structure and Mode-of-Action to Rational Design Concepts.

[17] Crimmin S., Grab S., Greenwood N., Jordon Z., Quirin S., Tournier N. (2019). The Complexity of Compliance in Sample Management: A Review of Key Issues Impacting Small-Molecule and Biological Sample Management in Early Drug Discovery. SLAS Technol..

[18] Kind T., Fiehn O. (2017). Strategies for dereplication of natural compounds using high-resolution tandem mass spectrometry. Phytochem. Lett..

[19] Mohimani H., Gurevich A., Mikheenko A., Garg N., Nothias L.F., Ninomiya A., Takada K., Dorrestein P.C., Pevzner P.A. (2017). Dereplication of peptidic natural products through database search of mass spectra. Nat. Chem. Biol..

[20] Mohimani H., Gurevich A., Shlemov A., Mikheenko A., Korobeynikov A., Cao L., Shcherbin E., Nothias L.F., Dorrestein P.C., Pevzner P.A. (2018). Dereplication of microbial metabolites through database search of mass spectra. Nat. Commun..

[21] Schorn M.A., Alanjary M.M., Aguinaldo K., Korobeynikov A., Podell S., Patin N., Lincecum T., Jensen P.R., Ziemert N., Moore B.S. (2016). Sequencing rare marine *actinomycete* genomes reveals high density of unique natural product biosynthetic gene clusters. Microbiology.

[22] Carroll A.R., Copp B.R., Davis R.A., Keyzers R.A., Prinsep M.R. (2019). Marine natural products. Nat. Prod. Rep..

[23] Avalon N.E., Murray A.E., Baker B.J. (2022). Integrated Metabolomic-Genomic Workflows Accelerate Microbial Natural Product Discovery. Anal. Chem..

[24] Abdelrahman S.M., Dosoky N.S., Hanora A.M., Lopanik N.B. (2022). Metabolomic Profiling and Molecular Networking of Nudibranch-Associated *Streptomyces* sp. SCSIO 001680. Molecules.

[25] Slama N., Mankai H., Ayed A., Mezhoud K., Rauch C., Lazim H., Barkallah I., Gtari M., Limam F. (2014). *Streptomyces tunisiensis* sp. nov. a novel *Streptomyces* species with antibacterial activity. Antonie Van Leeuwenhoek.

[26] Ibrahim W.M., Olama Z.A., Abou-elela G.M., Ramadan H.S., Hegazy G.E., El Badan D.E.S. (2023). Exploring the antimicrobial, antiviral, antioxidant, and antitumor potentials of marine *Streptomyces tunisiensis* W4MT573222 pigment isolated from Abu-Qir sediments, Egypt. Microb. Cell Factories.

[27] Slama N., Mankai H., Limam F. (2021). *Streptomyces tunisiensis* DSM 42037 mediated bioconversion of ferulic acid released from barley bran. World J. Microbiol. Biotechnol..

[28] Heel A.J.V., Jong A.D., Montalbán-López M., Kok J., Kuipers O.P. (2013). BAGEL3: Automated identification of genes encoding bacteriocins and (non-)bactericidal posttranslationally modified peptides. Nucleic Acids Res..

[29] Erba E., Bergamaschi D., Ronzoni S., Faretta M., Taverna S., Bonfanti M., Catapano C.V., Faircloth G., Jimeno J., D’Incalci M. (1999). Mode of action of thiocoraline, a natural marine compound with anti-tumour activity. Br. J. Cancer.

[30] Hartkoorn R.C., Sala C., Neres J., Pojer F., Magnet S., Mukherjee R., Uplekar S., Boy-Röttger S., Altmann K.H., Cole S.T. (2012). Towards a new tuberculosis drug: Pyridomycin—nature’s isoniazid. EMBO Mol. Med..

[31] Kiefer A., Bader C.D., Held J., Esser A., Rybniker J., Empting M., Müller R., Kazmaier U. (2019). Synthesis of New Cyclomarin Derivatives and Their Biological Evaluation towards *Mycobacterium Tuberculosis* and *Plasmodium Falciparum*. Chemistry.

[32] Alvarez-Mico X., Jensen P.R., Fenical W., Hughes C.C. (2013). Chlorizidine, a cytotoxic 5H-pyrrolo[2,1-a]isoindol-5-one-containing alkaloid from a marine *Streptomyces* sp. Org. Lett..

[33] Igarashi M., Tsuchida T., Kinoshita N., Kamijima M., Sawa R., Sawa T., Naganawa H., Hamada M., Takeuchi T., Yamazaki K. (1998). Cremimycin, a novel 19-membered macrocyclic lactam antibiotic, from *Streptomyces* sp. J. Antibiot..

[34] Wang M., Carver J.J., Phelan V.V., Sanchez L.M., Garg N., Peng Y., Nguyen D.D., Watrous J., Kapono C.A., Luzzatto-Knaan T. (2016). Sharing and community curation of mass spectrometry data with Global Natural Products Social Molecular Networking. Nat. Biotechnol..

[35] Kameyama T., Takahashi A., Kurasawa S., Ishizuka M., Okami Y., Takeuchi T., Umezawa H. (1987). Bisucaberin, a new siderophore, sensitizing tumor cells to macrophage-mediated cytolysis. I. Taxonomy of the producing organism, isolation and biological properties. J. Antibiot..

[36] Konetschny-Rapp S., Jung G., Raymond K.N., Meiwes J., Zaehner H. (1992). Solution thermodynamics of the ferric complexes of new desferrioxamine siderophores obtained by directed fermentation. J. Am. Chem. Soc..

[37] Meiwes J., Fiedler H.P., Zähner H., Konetschny-Rapp S., Jung G. (1990). Production of desferrioxamine E and new analogues by directed fermentation and feeding fermentation. Appl. Microbiol. Biotechnol..

[38] Jarmusch S.A., Lagos-Susaeta D., Diab E., Salazar O., Asenjo J.A., Ebel R., Jaspars M. (2021). Iron-meditated fungal starvation by lupine rhizosphere-associated and extremotolerant Streptomyces sp. S29 desferrioxamine production. Mol. Omics.

[39] García P.A., de Oliveira A.B., Batista R. (2007). Occurrence, biological activities and synthesis of kaurane diterpenes and their glycosides. Molecules.

[40] Li H., Sun B., Wang M., Hu X., Gao X., Xu S., Xu Y., Xu J., Hua H., Li D. (2018). Bioactive enmein-type 6,7-seco-ent-kaurane diterpenoids: Natural products, synthetic derivatives and apoptosis related mechanism. Arch. Pharm. Res..

[41] Becerril A., Álvarez S., Braña A.F., Rico S., Díaz M., Santamaría R.I., Salas J.A., Méndez C. (2018). Uncovering production of specialized metabolites by *Streptomyces argillaceus*: Activation of cryptic biosynthesis gene clusters using nutritional and genetic approaches. PLoS ONE.

[42] Wang W., Qiu Z., Tan H., Cao L. (2014). Siderophore production by actinobacteria. Biometals.

[43] Khasheii B., Mahmoodi P., Mohammadzadeh A. (2021). Siderophores: Importance in bacterial pathogenesis and applications in medicine and industry. Microbiol. Res..

[44] Shao M., Ma J., Li Q., Ju J. (2019). Identification of the Anti-Infective Aborycin Biosynthetic Gene Cluster from Deep-Sea-Derived *Streptomyces* sp. SCSIO ZS0098 Enables Production in a Heterologous Host. Mar. Drugs.

[45] https://www.bioinformatics.babraham.ac.uk/projects/trim_galore/.

[46] Nurk S., Bankevich A., Antipov D., Gurevich A.A., Korobeynikov A., Lapidus A., Prjibelski A.D., Pyshkin A., Sirotkin A., Sirotkin Y. (2013). Assembling single-cell genomes and mini-metagenomes from chimeric MDA products. J. Comput. Biol..

[47] Gurevich A., Saveliev V., Vyahhi N., Tesler G. (2013). QUAST: Quality assessment tool for genome assemblies. Bioinformatics.

[48] Rodriguez-R L.M., Gunturu S., Harvey W.T., Rosselló-Mora R., Tiedje J.M., Cole J.R., Konstantinidis K.T. (2018). The Microbial Genomes Atlas (MiGA) webserver: Taxonomic and gene diversity analysis of Archaea and Bacteria at the whole genome level. Nucleic Acids Res..

[49] Elmanzalawi M., Fujisawa T., Mori H., Nakamura Y., Tanizawa Y. (2025). DFAST_QC: Quality assessment and taxonomic identification tool for prokaryotic Genomes. BMC Bioinform..

[50] Chalita M., Kim Y.O., Park S., Oh H.-S., Cho J.H., Moon J., Baek N., Moon C., Lee K., Yang J. (2024). EzBioCloud: A genome-driven database and platform for microbiome identification and discovery. Int. J. Syst. Evol. Microbiol..

[51] Meier-Kolthoff J.P., Carbasse J.S., Peinado-Olarte R.L., Göker M. (2022). TYGS and LPSN: A database tandem for fast and reliable genome-based classification and nomenclature of prokaryotes. Nucleic Acids Res..

[52] Seemann T. (2014). Prokka: Rapid prokaryotic genome annotation. Bioinformatics.

[53] Blin K., Kim H.U., Medema M.H., Weber T. (2019). Recent development of antiSMASH and other computational approaches to mine secondary metabolite biosynthetic gene clusters. Brief Bioinform..

[54] Pluskal T., Castillo S., Villar-Briones A., Oresic M. (2010). MZmine 2: Modular Framework for Processing, Visualizing, and Analyzing Mass Spectrometry-Based Molecular Profile Data. BMC Bioinform..

[55] Shannon P., Markiel A., Ozier O., Baliga N.S., Wang J.T., Ramage D., Amin N., Schwikowski B., Ideker T. (2003). Cytoscape: A software environment for integrated models of biomolecular interaction networks. Genome. Res..

