# Supplementary material for: Genomic and Metabolomic Insights into Metabolites of a Streptomyces Isolate Associated with Chromodoris quadricolor, a Red Sea Nudibranch
Source: Mar Drugs. 2025 Oct 17;23(10):404. doi: 10.3390/md23100404 (PMC12565156; doi:10.3390/md23100404)
Supplement: Supplementary file 1 [file marinedrugs-23-00404-s001.zip › marinedrugs-3878989-supplementary.pdf]

Supplementary Tables

Table S1: Summary of the antiSMASH biosynthesis cluster prediction for the *Streptomyces* sp.

Region/ Node	Type	From	To	Most similar known cluster	Identity %	Bioactivity	Microorganism harbor this cluster	Ecological distribution	MIBiG BGC-ID
3	Terpene	6250	27173	Albaflavenone	100	Antibiotic	*Streptomyces coelicolor* A3	Soil	BGC0000660
4	NRPS,T1PKS, others	1	44729	Aurantimycin A	45	Antitumor and antibiotic	*Streptomyces aurantiacus JA 4570*	Soil	BGC0001519
6	Lanthipeptide	26033	42939	-	-	-	-	-	-
12	LAB, Bacteriocin	6396	29479	Chejuenolide A / B	7	-	*Hahella chejuensis*	Marine	BGC0001543
15	Terpene	581	27307	Hopene	92	Regulating membrane fluidity	*Streptomyces coelicolor* A3	Soil	BGC0000663
23	Betalactone	1	23782	-	-	-	-		-
26	NRPS	1	28654	Antimycin	81	Antibiotic and mycotoxin	*Streptomyces argillaceus*	Soil	BGC0001455
41	NRPS	1	22939	-	-	-	-	-	-
54	lassopeptide	1197	21662	Aborycin	100	Anti-Infective	*Streptomyces* sp. ZS0098	Marine	BGC0002015
71	Lanthipeptide	1	15218	Venezuelin	100	-	*Streptomyces venezuelae* ATCC 10712	Soil	BGC0000563
72	Siderophore	1	5873	-	-	-	-	-	-
80	T2PKS	1	19051	Curamycin	100	Spore pigment	*Streptomyces cyaneus*	-	BGC0000271
109	NRPS, T1PKS	1	16426	Caniferolide A-D	16	Attenuates Neuroinflammation	*Streptomyces caniferus*	Marine	BGC0001856
173	Bacteriocin	1	7550	Informatipeptin	57	-	*Streptomyces viridochromogenes* DSM	Soli	BGC0000518
241	Ecotine	1	7104	Ectoine	100	Irritation or inflammation	*Streptomyces anulatus*	Soil	BGC0000853
286	Ecotine	79	8131	Ectoine	100	Irritation or inflammation	*Streptomyces anulatus*	Soil	BGC0000853
292	Butyrolactone	1	7969	Scleric acid	17	-	*Streptomyces sclerotialus*	Soil	BGC0001771
308	Terpene	1	7672	Geosmin	100	-	*Streptomyces coelicolor* A3(2)	Soil	BGC0001181
368	Terpene	1	6367	Carotenoid	36	antioxidant activity	*Streptomyces avermitilis*	Soil	BGC0000633
384	Siderophore	1	5872	Grincamycin	8	Microbial and antitumor	*Streptomyces lusitanus*	Soil	BGC0000229
417	Other	1	5673	Himastatin	12	Antibiotic	*Streptomyces himastatinicus* ATCC 53653	Soil	BGC0001117
434	Siderophore	1	5532	Desferrioxamine E	100	Antitumor	*Streptomyces* sp. ID38640	Soil	BGC0001478
505	NRPS	1	4560	-	-	-	-	-	-
507	NRPS	1	4530	Antimycin	12	Antibiotic and mycotoxin	*Streptomyces argillaceus*	Soil	BGC0001455
527	NRPS	1	4313	-	-	-	-	-	-
578	Phenazine	1	3795	-	-	-	-	-	-
582	NRPS	1	3760	-	-	-	-	-	-
720	Lanthipeptide	1	2414	-	-	-	-	-	-
737	Butyrolactone	1	2340	γ-butyrolactone	66	Secondary metabolites regulator	*Streptomyces acidiscabies*	Soil	BGC0000850
774	NRPS	1	2119	-	-	-	-	-	-
830	T3PK	1	1750	Alkylresorcinol	66	Disinfectant- antibiotic	Streptomyces griseus subsp. griseus	Soil	BGC0000282
901	NRPS-Like	1	1335	Rhizomide A, B, C	100	Cytotoxic- pesticide	Paraburkholderia rhizoxinica HKI 454	Plant pathogenic fungus	BGC0001758
914	RiPP-like	1	1295	-	-	-	-	-	-

Table S2: Summary of the DeepBGC biosynthesis cluster prediction for the *Streptomyces* sp.

No.	ID	nucl_start	nucl_end	Product_activity	deepbgc_score	Align length	Product class
1	NODE_1_length_75760_cov_115.287679_0-56863.1	0	56863	-	0.73854	56863	Polyketide
2	NODE_2_length_61396_cov_108.491195_2-15067.1	2	15067	-	0.67833	15065	RiPP
3	NODE_2_length_61396_cov_108.491195_23466-28522.1	23466	28522	antibacterial	0.50982	5056	-
4	NODE_2_length_61396_cov_108.491195_55151-55379.1	55151	55379	antibacterial	0.52222	228	-
5	NODE_3_length_57431_cov_62.147005_17169-18537.1	17169	18537	antibacterial	0.53460	1368	Polyketide-Terpene
6	NODE_3_length_57431_cov_62.147005_34135-34396.1	34135	34396	antibacterial	0.54800	261	-
7	NODE_3_length_57431_cov_62.147005_44203-57430.1	44203	57430	antibacterial	0.75850	13227	-
8	NODE_4_length_44729_cov_81.550424_144-44728.1	144	44728	-	0.98864	44584	NRP-Polyketide
9	NODE_6_length_42939_cov_114.636317_173-42938.1	173	42938	antibacterial	0.91455	42765	RiPP
10	NODE_8_length_41410_cov_53.609670_2-10538.1	2	10538	antibacterial	0.61527	10536	RiPP
11	NODE_8_length_41410_cov_53.609670_37889-39751.1	37889	39751	antibacterial	0.57231	1862	Polyketide
12	NODE_9_length_40714_cov_40.016360_480-1683.1	480	1683	antibacterial	0.52719	1203	-
13	NODE_9_length_40714_cov_40.016360_15565-16783.1	15565	16783	antibacterial	0.55708	1218	-
14	NODE_9_length_40714_cov_40.016360_24703-25078.1	24703	25078	antibacterial	0.57059	375	-
15	NODE_9_length_40714_cov_40.016360_27211-29325.1	27211	29325	antibacterial	0.70132	2114	Polyketide
16	NODE_9_length_40714_cov_40.016360_30324-33213.1	30324	33213	antibacterial	0.60186	2889	Polyketide
17	NODE_10_length_38043_cov_50.127993_34193-34823.1	34193	34823	antibacterial	0.52225	630	-
18	NODE_11_length_37604_cov_60.681671_991-25030.1	991	25030	-	0.64624	24039	-
19	NODE_12_length_37432_cov_82.049698_16395-33057.1	16395	33057	antibacterial	0.64549	16662	RiPP
20	NODE_13_length_36805_cov_57.208899_33-1696.1	33	1696	antibacterial	0.58444	1663	-
21	NODE_14_length_35492_cov_46.124558_8679-35110.1	8679	35110	-	0.81429	26431	-
22	NODE_15_length_34845_cov_47.591134_2739-20327.1	2739	20327	-	0.76686	17588	Terpene
23	NODE_15_length_34845_cov_47.591134_24901-29453.1	24901	29453	antibacterial	0.60452	4552	-
24	NODE_22_length_31872_cov_63.313152_0-31872.1	0	31872	cytotoxic	0.90674	31872	-
25	NODE_23_length_31176_cov_51.874907_2-4493.1	2	4493	antibacterial	0.64735	4491	-
26	NODE_23_length_31176_cov_51.874907_10891-31050.1	10891	31050	antibacterial	0.85952	20159	-
27	NODE_24_length_31049_cov_72.737468_2466-17131.1	2466	17131	antibacterial	0.71298	14665	-
28	NODE_24_length_31049_cov_72.737468_21189-29674.1	21189	29674	antibacterial	0.60290	8485	

29	NODE_26_length_28654_cov_40.061100_0-28652.1	0	28652	-	0.99216	28652	NRP-Polyketide
30	NODE_29_length_27274_cov_42.689874_13432-18953.1	13432	18953	antibacterial	0.62298	5521	Polyketide
31	NODE_29_length_27274_cov_42.689874_20952-25402.1	20952	25402	antibacterial	0.65853	4450	-
33	NODE_31_length_26092_cov_35.871635_3819-4263.1	3819	4263	antibacterial	0.50384	444	-
34	NODE_31_length_26092_cov_35.871635_9203-21171.1	9203	21171	-	0.64101	11968	-
35	NODE_31_length_26092_cov_35.871635_23208-23592.1	23208	23592	antibacterial	0.59109	384	-
36	NODE_31_length_26092_cov_35.871635_24545-25031.1	24545	25031	antibacterial	0.50944	486	-
37	NODE_34_length_25366_cov_56.778240_0-1110.1	0	1110	antibacterial	0.55953	1110	-
38	NODE_34_length_25366_cov_56.778240_7444-7981.1	7444	7981	antibacterial	0.53366	537	-
39	NODE_38_length_25122_cov_62.585357_297-25120.1	297	25120	antibacterial	0.88084	24823	RiPP
40	NODE_41_length_24785_cov_70.031389_2-23994.1	2	23994	-	0.92880	23992	NRP
41	NODE_42_length_24591_cov_68.786462_9681-17392.1	9681	17392	antibacterial	0.59779	7711	-
42	NODE_43_length_24240_cov_56.424211_21422-22325.1	21422	22325	antibacterial	0.51671	903	-
43	NODE_50_length_22192_cov_74.247632_5455-6199.1	5455	6199	antibacterial	0.51551	744	-
44	NODE_50_length_22192_cov_74.247632_10389-22165.1	10389	22165	antibacterial	0.67804	11776	-
45	NODE_53_length_21662_cov_40.197818_0-21210.1	0	21210	antibacterial	0.91291	21210	RiPP
46	NODE_61_length_20861_cov_49.181441_0-13786.1	0	13786	antibacterial	0.71681	13786	-
47	NODE_63_length_20547_cov_57.971205_17503-19030.1	17503	19030	antibacterial	0.56133	1527	RiPP
48	NODE_65_length_20362_cov_47.475809_1-2457.1	1	2457	antibacterial	0.61737	2456	-
49	NODE_69_length_20084_cov_70.033773_206-2910.1	206	2910	antibacterial	0.59903	2704	-
50	NODE_69_length_20084_cov_70.033773_3444-4563.1	3444	4563	antibacterial	0.53972	1119	Saccharide-Terpene

Table S3: Summary of the napdos biosynthesis cluster prediction KS domain for the *Streptomyces* sp.

No.	Cand_id	Database match	Percent identity	Align length	E-value	BGC match	Domain class	Domain subclass
1	NODE_89_length_18058_cov_45.618593_3_4_30_452	StreptomycesFAS_KS01_FASII	93	429	2.46e-280	Streptomyces coelicolor FAS	type II FAS	no subclas
2	NODE_649_length_3039_cov_13.253434_6_0_109_539	splenocin_KS01_cisHybridKS	87	432	3.28e-269	splenocin	type I modular cis-AT	hybrid KS
3	NODE_80_length_19051_cov_49.575988_6_2_79_499	StreptomycesavermitilisSP_KS01_sporepigmentKSa	82	421	1.65e-249	Streptomyces avermitilis spore pigment	type II aromatic	spore pigment KSa
4	NODE_80_length_19051_cov_49.575988_4_7_142_550	StreptomycescollinusSP_KS02_sporepigmentKSb	79	411	1.23e-231	Streptomyces collinus spore pigment	type II aromatic	spore pigment KSb
5	NODE_666_length_2878_cov_42.456198_1_1_39_466	chlorothricin_KS03_cisAT	73	429	3.27e-229	chlorothricin	type I modular cis-AT	no subclass
6	NODE_4_length_44729_cov_81.550424_2_14_50_477	sanglifehrin_KS02_cisAT	73	428	5.44e-208	sanglifehrin	type I modular cis-AT	no subclass
7	NODE_108_length_16426_cov_54.678385_1_2_514_940	sceliphrolactam_KS03_cisAT	70	429	1.97e-195	sceliphrolactam	type I modular cis-AT	no subclass
8	NODE_4_length_44729_cov_81.550424_1_16_99_519	soraphen_KS01_cisloading	48	425	6.94e-111	soraphen	type I modular cis-AT	loading module
9	NODE_694_length_2649_cov_17.917922_2_0_216_576	StreptomycesFAS_KS01_FASII	48	368	1.43e-95	Streptomyces coelicolor FAS	type II FAS	no subclass

Table S4: *Summary of the napdos biosynthesis cluster prediction C domain for the Streptomyces* sp.

No.	ID	Database match	Percent identity	Align length	e-value	BGC product match	Domain class
1	NODE_4_length_44729_cov_81.550424_2_9_193_632	pristinamycin_C06_LCL	52	446	3.74E-134	pristinamycin	LCL
2	NODE_4_length_44729_cov_81.550424_2_9_1622_2090	thiocoraline_C02_epimerization	50	479	1.76E-125	thiocoraline	epimerization
3	NODE_4_length_44729_cov_81.550424_2_9_2107_2538	thiocoraline_C03_DCL	46	438	2.25E-116	thiocoraline	DCL
4	NODE_4_length_44729_cov_81.550424_3_3_18_456	cyclomarin_C02_LCL	53	445	7.37E-134	cyclomarin	LCL
5	NODE_4_length_44729_cov_81.550424_3_3_1089_1563	thiocoraline_C02_epimerization	49	476	2.91E-132	thiocoraline	epimerization
6	NODE_4_length_44729_cov_81.550424_3_3_1582_2008	thiocoraline_C03_DCL	45	433	2.95E-113	thiocoraline	DCL
7	NODE_530_length_4313_cov_22.815576_3_0_124_562	pristinamycin_C07_LCL	54	441	6.39E-136	pristinamycin	LCL
8	NODE_4_length_44729_cov_81.550424_2_10_5_441	cyclomarin_C04_LCL	49	439	2.45E-132	cyclomarin	LCL
9	NODE_585_length_3760_cov_20.959538_1_0_152_590	pristinamycin_C06_LCL	47	451	2.19E-105	pristinamycin	LCL
10	NODE_508_length_4530_cov_26.111969_4_1_44_475	bacillibactin_C01_starter	42	434	7.49E-104	bacillibactin	starter
11	NODE_26_length_28654_cov_40.061100_5_1_228_657	anabaenopeptilide_C04_LCL	38	431	1.09E-86	anabaenopeptilide	LCL
12	NODE_41_length_24785_cov_70.031389_3_0_64_468	pyoverdine_C08_LCL	43	415	3.02E-84	pyoverdine	LCL
13	NODE_371_length_6261_cov_23.921259_1_2_87_501	nostopeptolide_C08_LCL	36	431	3.20E-77	nostopeptolide	LCL
14	NODE_26_length_28654_cov_40.061100_6_2_241_674	anabaenopeptilide_C04_LCL	35	440	1.51E-67	anabaenopeptilide	LCL
15	NODE_776_length_2119_cov_33.625502_5_0_114_521	syringomycin_C09_LCL	32	433	6.46E-59	syringomycin	LCL
16	NODE_506_length_4560_cov_7.150237_2_0_151_532	microcystin_C03_modifiedAA	34	397	1.48E-55	microcystin	modified AA
17	NODE_108_length_16426_cov_54.678385_3_2_95_522	actinomycin_C01_starter	31	435	2.57E-46	actinomycin	starter
18	NODE_988_length_878_cov_18.905459_1_0_2_257	fengycin_C03_LCL	32	263	8.26E-35	fengycin	LCL

Table S5: Spectral information (LC-MS/MS) for compounds identified through GNPS. For all compounds: Ion source: LC-ESI; MS-Level: MS2; Instrument; qTOF; Ionization mode: Positive; MS Category: Experimental.

Compound	MoNA ID	Exact Mass	Precursor Type	Precursor m/z	Top 3-5 peaks
Desferrioxamine E	CCMSLIB00004695117	600.348	M+Na	623.337	623.337<br>423.2253<br>323.2240<br>106.1404<br>84.1856
Iso myristic acid	CCMSLIB00003135395	228.2089	M+H	229.2522	229.2522<br>187.2163<br>139.1749<br>89.1469<br>71.1859
Desferrioxamine X5	CCMSLIB00011905994	598.3690	M+H	599.3409	599.3409<br>399.2354<br>201.1653<br>181.1454<br>100.1565
Bisucaberin	CCMSLIB00005724304	400.232	M+H	401.2504	401.2504<br>201.1662<br>102.1711<br>84.1713<br>72.1797
Ferrioxamine D1+ Al (M-2H+Al)	CCMSLIB00005723664		M+H	627.3292	627.3292<br>467.2154<br>385.2146<br>285.1276<br>241.1466
Acyl_Desferrioxamine_C16	CCMSLIB00005716850	770.588	M+H	771.593	453.3555<br>401.2514<br>361.2631<br>319.2556<br>201.1660
Kaurane	CCMSLIB00005719951	272.285	M+H	273.2855	273.2855<br>217.2334<br>149.1913<br>107.1627<br>95.1694
Omega.-3 Arachidonic acid ethyl	CCMSLIB00003139185	332.272	M+H	333.279	331.2830<br>217.2338<br>149.1910<br>81.1626
Deferrioxamine H	CCMSLIB00000845582	460.253	M+H	461.267	461.2668<br>243.1665<br>201.1658<br>144.1621<br>102.1711

Table S6: The sequence alignment of the leader/core regions within the cluster.

	Leader	Core	Putative macrolactam
Peptide 53	MTAIYEPPALQEIGDFDELTK CLGIGSCNDFAGCGYAVVCFW	CLGIGSCNDFAGCGYAVVCFW	CLGIGSCND
MS-271	MGQLPHAGGTVRPVTSVTDHRPNKVGLTDPKREDIMSAIYEPPMLQEVGDFEELTK CLGVGSCNDFAGCGYAIVCFW	CLGVGSCNDFAGCGYAIVCFW	CLGVGSCND
Aborycin	MTAIYEPPALQEIGDFDELTK CLGIGSCNDFAGCGYAVVCFW	CLGIGSCNDFAGCGYAVVCFW	CLGIGSCND
Siamycin	MSAIYEPPMLQEVGDFEELTK CLGVGSCNDFAGCGYAIVCFW	CLGVGSCNDFAGCGYAIVCFW	CLGVGSCND

Supplementary Figures

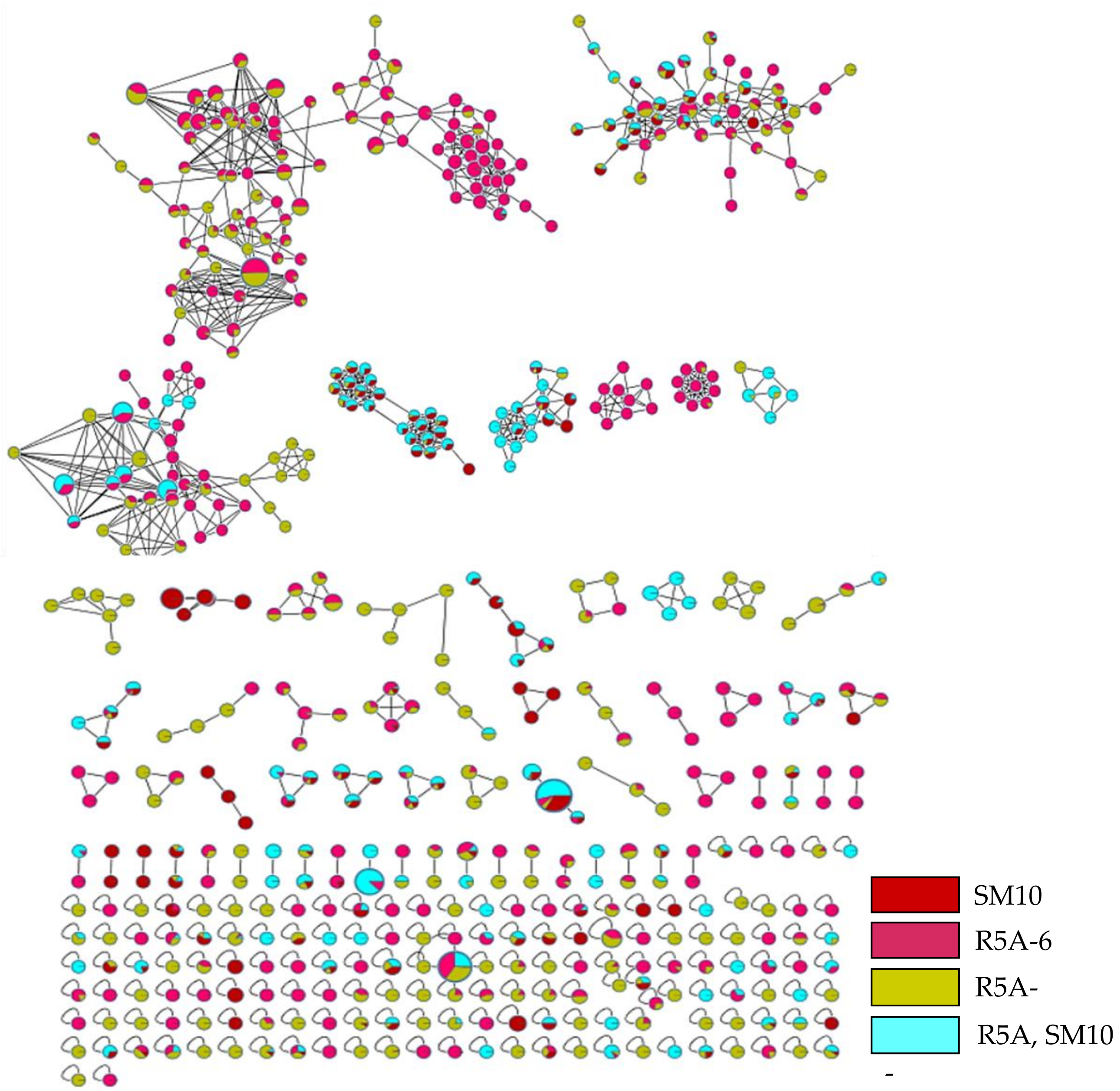

Figure S1. Molecular networking of metabolites produced by *S. tunisiensis* generated by Cytoscape version 3.7.1. (GNPS cosine cutoff 0.7)

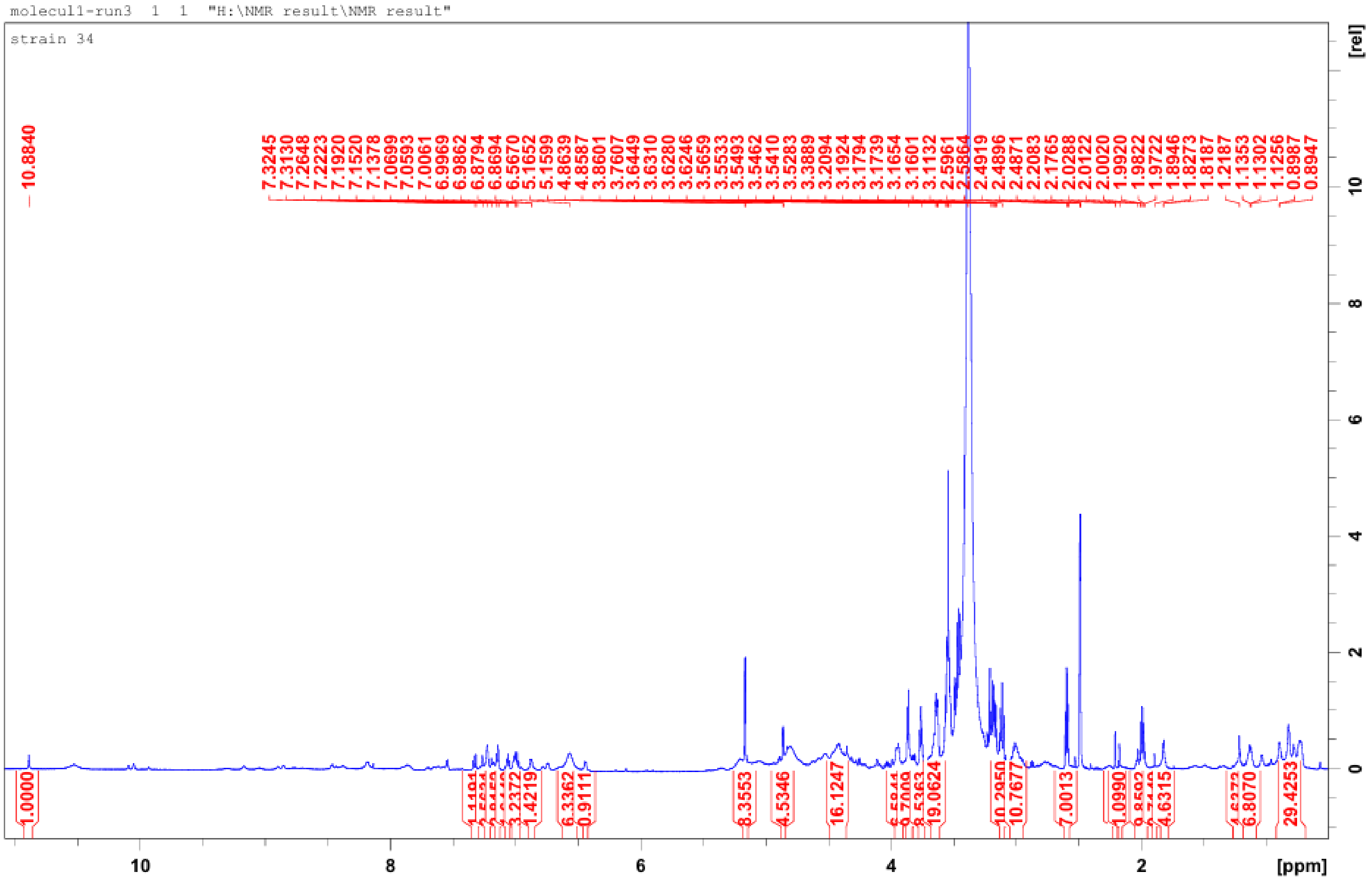

Figure S2. ^{1}H NMR spectra for the isolated molecule (Aborycin).

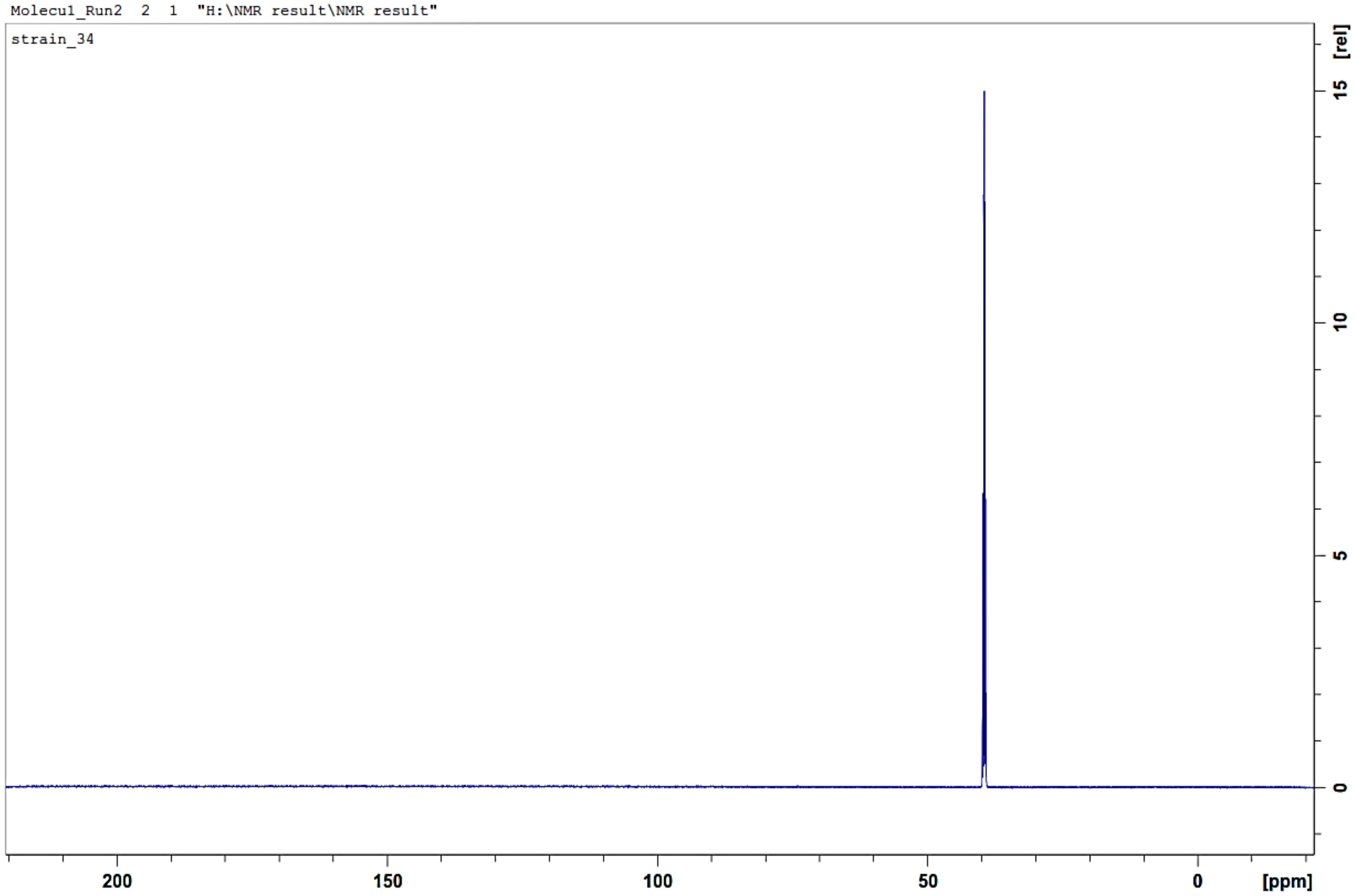

Figure S3. ^{13}C NMR spectra for the isolated molecule (Aborycin).

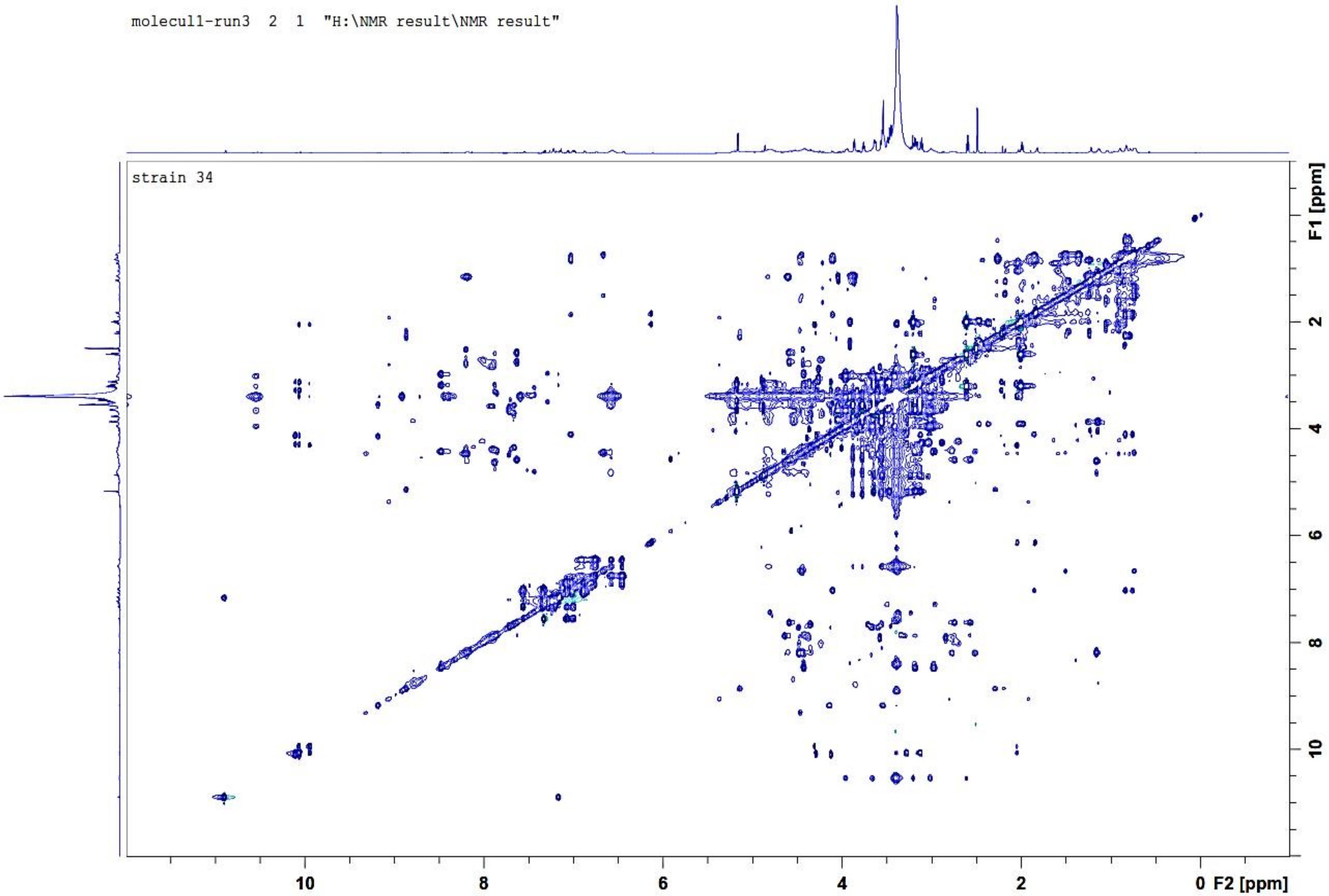

Figure S4. COSY NMR spectra for the isolated molecule (Aborycin).

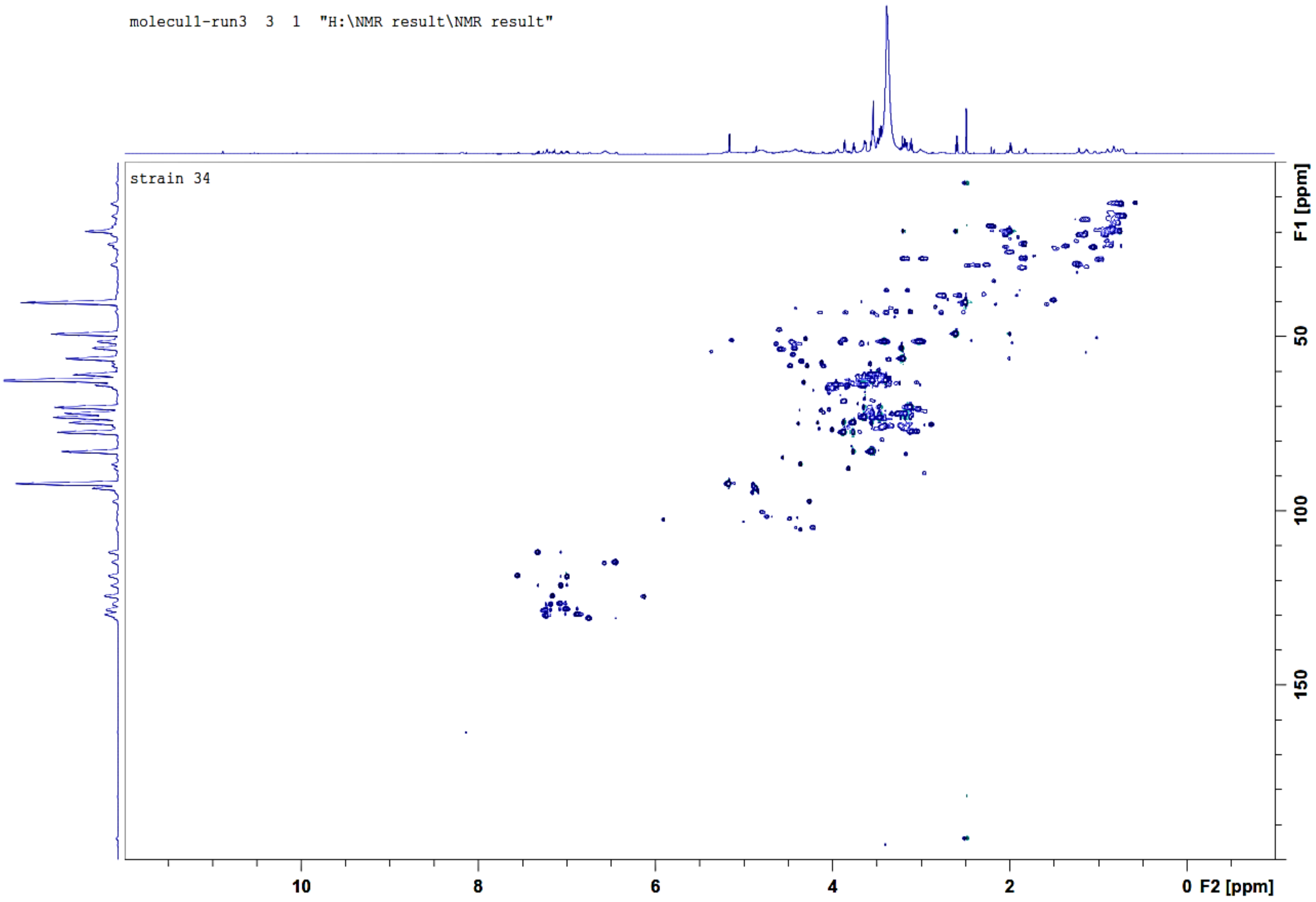

Figure S5. HSQC NMR spectra for isolated molecule (Aborycin).

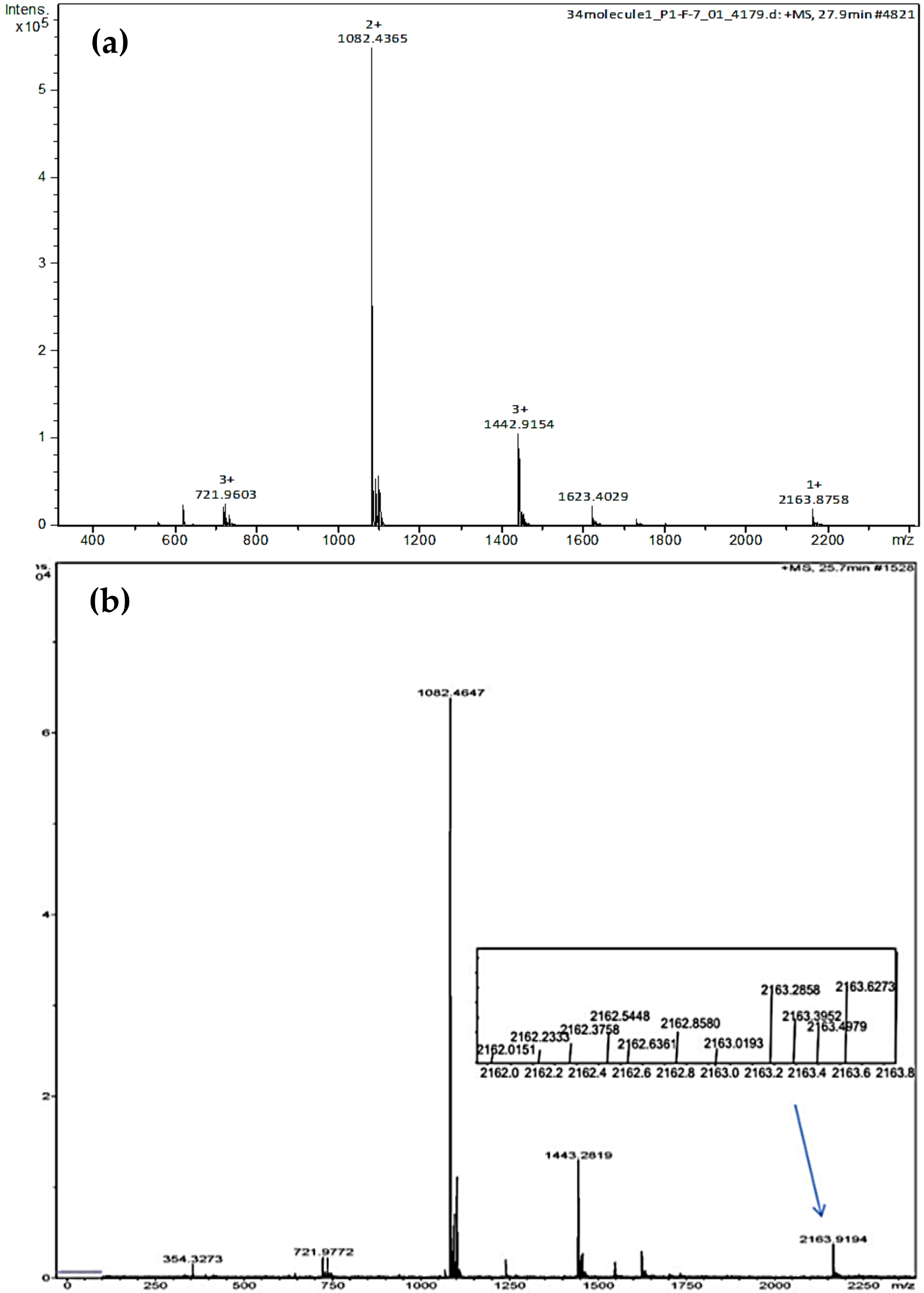

Figure S6. LC-MS comparative analysis of lassopeptide; (**a**) studied molecules accumulated in the culture of *Streptomyces tunisiensis*. (positive mode) and (**b**) aborycin accumulated in culture of *Streptomyces coelicolor* [44].
